# Evolving Trends in the Management of Diabetic Foot Ulcers: A Narrative Review

**DOI:** 10.7759/cureus.65095

**Published:** 2024-07-22

**Authors:** Omo A Ogbeide, Stephanie I Okeleke, Joy C Okorie, Jagshak Mandong, Adetayo Ajiboye, Oluseyi O Olawale, Fuseina Salifu

**Affiliations:** 1 General Medicine, Nuffield Health Plymouth Hospital, Plymouth, GBR; 2 Public Health, East Tennessee State University, Johnson, USA; 3 Family and Community Medicine, Nissi Family Medicine, Austell, USA; 4 Medicine, King Fahad Hospital Hofuf, Hofuf, SAU; 5 Internal Medicine, Birmingham and Solihull Mental Health NHS Foundation Trust, Birmingham, GBR; 6 Family and Community Medicine, Walden University, Minneapolis, USA; 7 General Medicine, Ridge Hospital, Accra, GHA

**Keywords:** negative pressure wound therapy, type 2 diabetes mellitus (dm), maggot debridement therapy, pad (peripheral artery disease), dfu (diabetic foot ulcer), diabete mellitus

## Abstract

The prevalence of diabetic foot ulcers (DFUs) is projected to increase worldwide, which necessitates a review of the current management principles and the development of new approaches to care. The principles of management involve proper glycemic control, infection control, pressure redistribution, wound care debridement, and revascularization. Other modalities of management, such as hyperbaric oxygen therapy and negative wound pressure therapy, are also being explored. While some aspects of DFU care lack high-quality evidence, a multidisciplinary approach incorporating these evolving trends has the potential to improve outcomes and prevent lower extremity amputations in this challenging condition. This review highlights the need for further research to establish definitive treatment protocols for optimal DFU management.

## Introduction and background

Diabetic foot ulcer (DFU) is a long-term complication of diabetes mellitus (DM), a medical condition characterized by hyperglycemia that can lead to damage to various organs in the body. Widely considered an epidemic, DM is on the rise worldwide. DM is predicted to affect approximately 550 million people worldwide, with that figure expected to rise to 578 million by 2030 and 700 million by 2045 [1]. The prevalence of DM has increased over recent decades [1]. The CDC reports that about 38.4 million Americans, or roughly 11.6% of the population, suffer from DM, with actual estimates likely to be higher [2]. In the United States, the total cost of managing diabetes was estimated to be $413 billion in 2022, while between 2012 and 2022, $10,179-$12,022 were spent per person on managing diabetes [2]. The problem of DFUs was first described halfway through the 19th century by Marchal de Calvi in 1852 and Thomas Hodgkin in 1854, who hypothesized that there was an association between diabetes and gangrene of the foot [3]. Frederick Treves, at the end of the 19th century, was the first to carry out debridement of a diabetic ulcer [3]. A demographic change worldwide, increasing urbanization and physical inactivity as well as obesity, has seen an increase in diabetes-related complications such as DFUs [1].

The development of DFUs is often influenced by various factors, such as neuropathy, peripheral arterial disease (PAD), and compromised immune function. If left unaddressed, DFUs can lead to severe consequences like infection, gangrene, and potential amputation [4]. DFUs impact around 15% of individuals with diabetes during their lifetime. Diabetic foot infections affect approximately 50% of people with DFUs, with 20% of these requiring hospitalization. It is estimated that 15-20% of those hospitalized will eventually require lower extremity amputation [4].

The management of DFUs typically involves a collaborative effort among various healthcare professionals, including podiatrists, endocrinologists, vascular surgeons, and infectious disease specialists [5]. The key components of management encompass comprehensive wound care through regular debridement, the application of appropriate dressings, and alleviating pressure from the affected area with specialized footwear or orthotics [6]. The increasing prevalence of DFUs has made it necessary to develop novel strategies for addressing this growing issue. This review aims to explore current strategies in the management of DFUs and highlight new trends.

## Review

Pathophysiology

Despite numerous preventive interventions, diabetes remains a prominent factor contributing to lower limb amputations from nontraumatic injuries in the United States [7]. Foot ulcers and infections significantly contribute to the disease burden among patients with DM. Known pathogenic factors responsible for DFUs include neuropathy, PAD, and immune dysfunction, all of which have an underlying connection to the hyperglycemic state of diabetes. Various pathogenetic concepts have been associated with hyperglycemia, such as dysfunction in the metabolism of polyol and myoinositol, defects in axonal transport, oxygen-free radical generation, nonenzymatic glycosylation of neuronal structure and transport proteins, and endoneurial-microvascular loss with subsequent ischemia [8]. Underlying foot anomalies, such as bone spurs, hypertrophic bursae, and structural abnormalities, have also been tied to DFUs [9].

Neuropathy

Diabetic neuropathy can manifest as diffuse neuropathy, including distal symmetrical polyneuropathy, autonomic neuropathy, mononeuropathy, radiculopathy, and/or polyradiculopathy [10]. Peripheral sensory neuropathy leads to a loss of protective sensation, rendering patients unaware of minor foot injuries [10,11]. Disordered proprioception can cause abnormal weight-bearing during ambulation, leading to callus formation [10]. Both motor and sensory neuropathies contribute to atrophy and structural changes in the foot, such as claw deformity and hammer toe [10,12]. Autonomic neuropathy disrupts nerve function in the feet, altering blood flow and causing anhidrosis, which can result in dry and cracked skin. Frequent weight-bearing impacts on the foot can cause minor injuries and inflammation, leading to bleeding beneath calluses, which may develop into full-thickness ulcers when the callus is removed [4].

PAD

In patients with diabetes, the distribution of PAD is distinct, preferentially affecting the posterior and anterior tibial arteries, with less frequent involvement of the superficial femoral and popliteal arteries [12]. Occlusions in these arteries can reduce blood flow to the foot, potentially resulting in ischemic ulcers and infection.

Immune Dysfunction

Poor glycemic control can cause immune dysfunction, impairing leukocyte function and complement activity, which in the presence of an ulcer can promote bacterial growth [12]. Additional immune changes, such as increased T lymphocyte apoptosis, have been observed to inhibit wound healing in patients with DFU [13].

Clinical manifestations and evaluation

The clinical manifestations of DFUs should be assessed following a detailed clinical history for a patient with DM, medication history, and associated complications. Signs of inadequate vascular perfusion and neuropathy should also be assessed [14,15]. It is important to assess DFU in every patient being reviewed for DM, and examinations should be carried out in the presence of adequate lighting. Ulcer characteristics such as size, depth, look, and location should be properly recorded [14,16].

Clinical findings can be categorized based on the following signs and symptoms:

Neuropathy

There are three types of neuropathy: sensory, motor, and autonomic. Each increases the likelihood of developing a foot ulcer [17]. Sensory neuropathy can present with either reduced sensation or increased sensation. Findings in the examination may include accidental burns and wounds. Some patients present with radicular pain [14]. Motor neuropathy can also cause atrophy of the intrinsic muscles in the foot. Hammer toe and claw toe abnormalities are caused by increased joint extension and flexion at the foot. Assessment of footwear is crucial since it can contribute to the formation of foot ulcers [14,17]. Autonomic neuropathy can result in an accelerated risk of skin atrophy, along with dry or overly moist skin. Brittle toenails, arteriosclerosis, Charcot’s foot, and neuropathic edema can also be observed [14,17].

PAD

During cardiovascular system examination, the pulse of distal arteries of the lower limbs, such as the popliteal, posterior tibial, and dorsalis pedis, should be assessed for absence or reduction in pulse volume. Claudication, hair loss, and evidence suggesting imminent ischemia such as pale, thin, shiny, or cold skin also contribute to poor wound healing [14].

Diabetic Foot Infection

Infection can manifest as either localized superficial skin involvement or deeper skin structures that have progressed beyond the location of the initial damage. Infections like this can spread to joints, bones, and systemic circulation [15].

Diabetic foot infections are severe due to background immunosuppression. If patients are not properly managed, they can present with symptoms of widespread infection such as cellulitis, osteomyelitis, or sepsis, which in severe cases can end in major or minor amputations or death [17].

Just like any other disease, a detailed history, physical examination, and investigations are important steps in the management of DFUs. Take a comprehensive history, inquiring about symptoms of PAD, neuropathy, the presence of comorbidities, glycemic control, previous foot ulceration or amputation, smoking history, and information about footwear [18]. A clinical examination should include inspecting the foot for ulcers and their location, foot deformities, the presence of calluses, surrounding skin changes, loss of sensation, palpable pulses, and any signs suggestive of infection [18].

Assess for neuropathy using a monofilament test to check for light pressure sensation at 12 different sites [18]. Use a 128-Hz tuning fork to test for vibratory sense and temperature perception [18]. Perform a pinprick test and assess deep tendon reflexes. The severity of sensory loss can be scored using the modified Neuropathy Disability Score (NDS) to determine the risk of further ulceration (Table 1). A score of 10 indicates total loss of sensation, while an NDS of ≥6 indicates a higher risk of foot ulcers [18].

**Table 1:** Modified NDS NDS, Neuropathy Disability Score

Test	Score	
	Right foot	Left foot
Vibration sensation (using a 128-Hz tuning fork)	Normal = 0; abnormal = 1	Normal = 0; abnormal = 1
Temperature sensation (using a tuning fork with a beaker of ice or warm water)	Normal = 0; abnormal = 1	Normal = 0; abnormal = 1
Pinprick test	Normal = 0; abnormal = 1	Normal = 0; abnormal = 1
Achilles reflex	Present = 0; present with reinforcement = 1; absent = 2	Present = 0; present with reinforcement = 1; absent = 2
	NDS total out of 10	

Laboratory investigations should include hemoglobin A1C, glucose level, complete blood count with differential, serum electrolytes, creatinine, blood urea nitrogen, urinalysis, prealbumin level, erythrocyte sedimentation rate, lipid, and clotting profile [12,19]. In addition to other tests, a septic workup, such as wound cultures, blood cultures, and a plain radiograph, is mandatory for ulcers showing signs of infection [18]. Some studies suggest that taking specimens from deep tissues is more reliable for guiding antimicrobial therapy than superficial specimens [20].

A number of imaging modalities help in the diagnosis of osteomyelitis, which is often present in many DFUs [21]. These include X-rays (which have low sensitivity), three-phase bone scans (high sensitivity but less reliable in severe PAD), MRI (high sensitivity and specificity), radiolabeled scintigraphy of the foot, and single-photon emission computed tomography (SPECT) scans [12, 21]. A SPECT scan can provide a more definitive diagnosis of osteomyelitis than an MRI, as the latter may sometimes yield false-positive results [9].

Noninvasive arterial testing of the limb should be performed on patients without detectable pulses to screen for the presence of PAD [12]. These include measurements of transcutaneous oxygen pressure (TcpO2), ankle-brachial index (ABI), and toe systolic pressure [12,18]. TcpO2 of 30 mmHg or greater, ankle systolic pressure of 65 mmHg or greater, and toe systolic pressure of 40 mmHg or greater have been shown to be beneficial for ulcer healing [12]. However, there are some disagreements over the sensitivity and specificity of the noninvasive tests, and these tests have been criticized for underestimating the extent of arterial insufficiency [22]. If there is a high level of suspicion for lower extremity ischemia, duplex ultrasonography may be utilized to assess the patency of arteries and ascertain the extent and severity of the disease, or arteriography in the case of severe stenosis or inconclusive results [12].

Management of DFU

Glycemic Control

The evidence on the role of glycemic control in the management of DFUs is still unclear, with conflicting findings from various studies. It is believed that poor glycemic control is associated with an increased risk of lower extremity amputations. However, very few studies have shown a direct correlation between glycemic control and wound healing [23]. Despite the dearth of studies supporting intensive glycemic control, it is generally thought that normoglycemia facilitates the healing of DFU by improving all phases of wound healing. Hyperglycemia impairs neutrophil phagocytosis, abnormal migration of keratinocytes, and impaired collagen synthesis, which in turn can impede wound healing [23].

There is now greater emphasis on a patient-centered approach to achieve euglycemia in people with T2DM [24]. While weight loss, diet, and lifestyle changes are invaluable in achieving euglycemia, a lot of patients will require antidiabetic medications. Recent studies have shown that the early institution of more than one agent produces better glycemic control, thereby preventing DFU [24]. As patients with T2DM sometimes present with DFU as their initial presentation, achieving glycemic control in these patients is even more important. Insulin is the preferred agent for achieving euglycemia in hospitalized patients [25]. Insulin administration should be closely monitored so as to avoid hypoglycemia [25]. Newer agents being used to treat T2DM include SGLT2 inhibitors, GLP-1 agonists, and combined GLP-1 and GIP agonists. SGLT2 inhibitors have been shown to improve cardiovascular and renal outcomes in patients with T2DM. The CANVAS program showed that even though canagliflozin, an SGLT2 inhibitor, is linked with a reduced risk of cardiovascular disease and the progression of albuminuria, it is associated with a high risk of amputation, especially of the toe and metatarsal [26].

Wound Debridement

Wound debridement is often the initial stage and plays a vital role in the healing of DFUs. A favorable environment characterized by absent unviable tissues, features of infection, and contaminants is needed for proper wound care; this is achieved by adequate wound debridement [21,27]. In situations where there is a need for wound culture, it is to be taken after debridement. If there is a need for wound cleansing, saline solutions rather than antiseptics should be used before taking wound culture. This helps to reduce false negative results [28].

The Infectious Diseases Society of America (IDSA) and the Wound Healing Society propose employing sharp debridement devices rather than topical debridement treatments. Sharp debridement has been demonstrated to be successful in various clinical trials, despite the scarcity of cumulative data [29]. Debridement significantly reduces bacteria load, serves as a barrier for antibiotics, and stimulates contraction and wound epithelialization [28,29].

Cutting-edge technologies are considering the use of the following:

Maggot debridement therapy: This involves the clinical utilization of sterilized medical worms such as the Australian sheep blowfly *Lucilia cuprina *or green bottle fly *Lucilia sericata*. These are grown under well-controlled laboratory conditions for the debridement of diabetic ulcers if surgical debridement is not accessible or feasible [27,30].

Enzymatic debridement: Exogenous enzyme products, such as collagenase, are used to digest nonviable tissue rather than depending entirely on naturally synthesized wound enzymes like matrix metalloproteinases [28,30].

Autolytic debridement: This method requires keeping the wound moist with hydrogel, which may stimulate the wound’s own generation of endogenous enzymes to self-digest dead tissue. Alginates and hydrocolloids can also be used. Foam and film are also commonly used. The use of honey and some other dressings can help with autolytic debridement and healing [28,30].

Wound Dressing

It is widely accepted that the purpose of a dressing should be to establish a moist environment that promotes the creation of excellent granulation tissue, autolytic processes, and angiogenesis, as well as faster recruitment of epidermal cells over the wound base [29,30].

Numerous dressing options are available and continue to be explored, but there is little evidence to support any particular method. The choice of dressing should be based on the unique circumstances of each case [27,30]. Broad guidelines for managing DFUs include using hydrogels during the debridement stage, low-adherent dressings to maintain moisture during the granulation stage, and low-adherent dressings for the epithelial development stage [27,30].

Dressings are typically replaced daily or at intervals that prevent disruption of the healing process and allow for adequate granulation. Common types of dressings are classified as follows: Open dressings include, primarily, gauze, which is typically moistened with saline before placing it into the wound [27,30]. Semi-open dressings often consist of fine mesh gauze soaked with petroleum, paraffin wax, or other ointment. This first layer is covered by an additional dressing of absorbent gauze and cushioning, followed by a final layer of other adhesives [27,30]. Semi-occlusive dressings have a range of occlusive qualities, along with absorbent capabilities and antimicrobial activity. Semi-occlusive dressings utilize films and foams. In addition, the use of alginates and hydrocolloid dressings is also commonly practiced [27,30].

Infection Control

Infection has a major role in the progression of complications associated with DFUs. This role is more commonly associated with bacteria such as Gram-positive bacteria like *Staphylococcus aureus *and beta-hemolytic *Streptococci*, as well as gram-negative bacteria like *Pseudomonas*. These bacteria create biofilms that develop resistance to antimicrobial agents and result in chronic inflammation [31].

DFUs can have serious effects, including a lower quality of life, a life-threatening infection, and the need for amputation. Infection hinders cellular regeneration and delays the healing process of DFU. Therefore, it is crucial to prioritize infection control and prevention to facilitate proper blood supply and minimize tissue inflammation. Evidence of infection in DFU can be observed through inflammatory symptoms such as erythema, purulence, warmth, and a bad-smelling odor [29,31].

The IDSA recommends treating DFU with broad-spectrum antibiotics if there are two or more inflammatory symptoms. A culture should be conducted before commencing antibiotics, and the treatment strategy should be limited based on the culture results. The use of narrow-spectrum antibiotics is recommended after culture results [29].

Pressure Redistribution

The goal of pressure redistribution is to alleviate plantar pressure, which slows wound healing and prevents further complications by shifting the body’s weight away from the foot [32]. Offloading can be achieved through different methods, including the use of boots, total contact casts (TCCs), nonremovable high-knee devices, and orthotic walkers.

TCC is well recognized as an efficient approach for dispersing the entire load borne by the foot. However, it should not be utilized when there is an infection present [33]. The International Working Group on the Diabetic Foot considers nonremovable high-knee devices an efficient technique of off-loading pressure in DFU, especially in settings where the skill of casting is unavailable [29,33]. Orthotic walkers not only give stability but also limit joint mobility and control foot deformities while easing the burden and evenly spreading the foot’s pressure [34].

Revascularization

Revascularization is a very effective management strategy for restoring blood flow in DFU once ischemia is diagnosed. The treatment’s goal is to salvage the limb while simultaneously treating the ulcer to avoid the need for amputation [35]. The benefits of revascularization should outweigh its risks [36].

Revascularization techniques, whether direct or indirect, aim to improve blood flow to the foot through perfusion. Direct revascularization often focuses on the artery that supplies the tissue. While indirect revascularization selects the most appropriate artery, regardless of its anatomical location [37].

Revascularization procedures include angioplasty, with or without stenting, and surgical bypass. Sometimes, a combination of the two strategies can be used with the same patient [36]. Percutaneous transluminal angioplasty is the primary choice of treatment and is widely used because it offers several advantages to the patient, including greater tolerability and repeatability in the event of re-occlusion. The goal of the angioplasty is to achieve straight line flow from the aorta to the foot arteries with a patent dorsalis pedis or plantar arch [35].

Traditionally, arterial bypass surgery has been the primary therapeutic option due to its high long-term patency and limb salvage rates. Surgical challenges include limited availability of long vein grafts, infection near distal anastomosis sites, and patient comorbidities. Multi-vessel blockage, limited arteries and inflow, and poor distal runoff may interfere with surgical revascularization outcomes [35].

Hyperbaric Oxygen Therapy

Hyperbaric oxygen therapy has shown promise in managing DFU in several studies. In this therapy, patients are exposed to 100% oxygen in a hyperbaric chamber [38]. It is yet to be widely used in the management of DFU, as the overall evidence of its benefit has yet to be demonstrated. It has been shown to prevent amputations and promote healing in patients with severe DFU [38,39].

Negative Pressure Wound Therapy

Negative pressure wound therapy is a form of therapy that promotes wound healing by exerting negative pressure on the wound surface via a mechanical pump. Reviews have shown that it is unclear whether negative pressure wound therapy should be widely adopted in the management of DFU [40].

Skin Substitutes

The use of skin substitutes in managing DFUs has been the subject of intense study. Skin substitutes act by promoting the proliferation of extracellular matrices as well as limiting the impact of trauma and infection on the wound surface [38]. A systematic review done in 2016 showed that skin substitutes, in addition to standard care, are capable of achieving complete ulcer closure compared to standard care alone [41]. Skin substitutes, together with standard care, were also found to be less likely to result in lower extremity amputations compared to standard care alone [41]. These studies were, however, noted to lack long-term follow-up, making it difficult to ascertain the rate of ulcer recurrence following the use of skin substitutes [41].

Topical Growth Factors

Topical growth factors are being explored in the management of DFUs, one of which is becaplermin, a recombinant platelet-derived growth factor approved by the FDA for the treatment of DFU extending to and beyond the subcutaneous tissue in patients without ischemia [42]. It has been shown in studies to speed up healing time and reduce the risk of lower extremity amputation [42]. This medication is associated with an increased risk of cancer [38]. It is yet to be widely adopted in managing DFU, despite being shown to be cost-effective when used in conjunction with standard care compared to standard care alone [43].

## Conclusions

The management of DFUs has significantly advanced over time, with a clear trend toward a compact, patient-centered, multidisciplinary approach. Optimal glycemic control, infection control, pressure redistribution, re-vascularization, wound care, and debridement remain key to preventing and managing DFUs. Emerging trends like hyperbaric oxygen therapy, negative wound pressure therapy, skin substitutes, and growth factor therapy are promising, and there is a need for further randomized and observational studies. Together with patient participation and education, the evidence suggests a positive trajectory toward improved quality of life for patients and a reduction in the healthcare burden associated with DFUs.
